# Surveillance of *Enterobacter cloacae* complex colonization and comparative analysis of different typing methods on a neonatal intensive care unit in Germany

**DOI:** 10.1186/s13756-022-01094-y

**Published:** 2022-04-01

**Authors:** Andreas F. Wendel, Daniel Peter, Frauke Mattner, Michael Weiss, Marc Hoppenz, Sophia Wolf, Baris Bader, Silke Peter, Jan Liese

**Affiliations:** 1 Institute of Hygiene, Cologne Merheim Medical Centre, University Hospital of Witten/Herdecke, Ostmerheimer Strasse 200, 51109 Cologne, Germany; 2 Division of Hygiene and Environmental Medicine, Department of Human Medicine, Faculty of Health, Witten/Herdecke University, Witten, Germany; 3 Department of Neonatology and Pediatric Intensive Care Medicine, Children’s Hospital, Cologne, Germany; 4 Institute of Medical Microbiology and Hygiene, University Hospital Tübingen, Tübingen, Germany; 5 German Center for Infection Research (DZIF), Partner Site Tübingen, Tübingen, Germany

**Keywords:** *E. cloacae* complex, Neonatal intensive care unit, Bacterial typing, Neonatal colonization screening

## Abstract

**Background:**

*Enterobacter cloacae* complex is a group of common opportunistic pathogens on neonatal intensive care units. Active microbiological screening to guide empirical antimicrobial treatment or to detect transmission events is recommended in high-risk preterm neonates. A rise in colonization with *E. cloacae* complex was observed in a German perinatal centre. The aim of this study was to evaluate the performance of different typing techniques using whole genome sequencing (WGS) as a reference.

**Methods:**

*Enterobacter cloacae* complex isolates from clinical and screening specimens with an epidemiological link to the neonatal intensive care units were further assessed. Identification and antibiotic susceptibility testing was performed by a combination of VITEK2 (bioMérieux) and MALDI-TOF (Bruker Daltonics), followed by RAPD/rep-PCR and PFGE (*Xba*I). Retrospectively, all isolates were analyzed by Fourier-transform infrared (FTIR) spectroscopy (IR Biotyper, Bruker Daltonics). Whole genome sequencing with SNP-based clustering was used as the reference method. Furthermore, resistome analysis, sequence type and species identification were derived from the WGS data. Transmission analysis was based on epidemiological and typing data.

**Results:**

Between September 2017 and March 2018 32 mostly preterm neonates were found to be colonized with *E. cloacae* complex and 32 isolates from 24 patients were available for further typing. RAPD/rep-PCR and PFGE showed good concordance with WGS whereas FTIR displayed mediocre results [adjusted rand index (ARI) = 0.436]. A polyclonal increase and two dominant and overlapping clonal clusters of two different *E. hormaechei* subspecies were detected. Overall, four different species were identified. Genotyping confirmed third-generation cephalosporin resistance development in isolates of the same patient. During the six-month period several infection prevention interventions were performed and no *E. cloacae* complex isolates were observed during the following months.

**Conclusions:**

Interpretation of the microbiological results alone to detect transmission events is often challenging and bacterial typing is of utmost importance to implement targeted infection control measures in an epidemic occurrence of *E. cloacae* complex. WGS is the most discriminatory method. However, traditional methods such as PFGE or RAPD/rep-PCR can provide reliable and quicker results in many settings. Furthermore, research is needed to quickly identify *E. cloacae* complex to the species level in the microbiological laboratory.

## Background

*Enterobacter cloacae* complex comprises several closely related *Enterobacter* species that are difficult to differentiate by standard microbiological methods such as biochemical profiling or mass spectroscopy. Members of this genetically diverse group are described in the environment or as commensals of the animal and human gut. Moreover, *E. cloacae* complex has emerged as an important facultative pathogen and significant cause of hospital-acquired infections. Special attention has been paid to infections with multidrug-resistant *E. cloacae* complex. Patient-to-patient or environment-to-patient transmissions can lead to outbreaks in the hospital setting [1–4]*.*

In preterm neonates, early gut colonization with *E. cloacae* complex is common [5, 6]. At the same time, *E. cloacae* complex is one of the most common causes of outbreaks in this specialized setting and can cause severe infections [7]. For this reason, microbiological colonization screening for *E. cloacae* complex is officially recommended in high-risk neonates with a gestational weight below 1500 g in Germany (irrespective of the antibiotic susceptibility profile and amongst other relevant Gram-negative and Gram-positive bacteria) [8, 9]. However, interpretation of this data is often challenging. If transmission is suspected on the basis of spatio-temporal relationship of the isolates/patients, quick, reliable and discriminatory genotyping is of utmost importance to implement targeted infection control measures [10].

Here we report of a sudden increase of patients colonized with *E. cloacae* complex on a neonatal intensive care unit and highlight the challenges and pitfalls related to different typing techniques applied.

## Methods

### Setting and screening strategy

The perinatal centre of the Children's Hospital of Cologne (neonatal level III care according to international classification [11]) includes a 10-bed neonatal intensive care unit (NICU) and another 16-bed paediatric (and neonatal) intensive care unit (PICU). The two units are housed in two separate hospitals; the NICU is located next to the maternity ward, whereas the PICU is located in the Children’s Hospital. In general, preterm neonates are hospitalized first on the NICU and thereafter released to the neonatal general ward in the Children’s Hospital. If surgery or other highly-specialized treatment is indicated, patients are transferred to the Children’s Hospital. The protocol of the German healthcare-associated infection surveillance for very low birthweight infants (NEO-KISS, < 1.500 g) and on intensive care units (ITS-KISS, PICU) was followed during the study period [12, 13]. A weekly microbiological screening (perianal and combined nasal/oropharyngeal swabs) was performed on all infants of the NICU and on the preterm neonates of the PICU and the neonatal general ward according to the German infection control guideline that includes screening for *E. cloacae* complex [8]. The number of patients colonized/infected with *E. cloacae* complex was assessed using the laboratory surveillance information system HyBASE® v.6 (epiNET AG, Bochum, Germany).

### Identification and susceptibility testing

Screening swabs were inoculated on Columbia blood agar, chocolate agar, McConkey agar (all Becton Dickinson, Heidelberg, Germany) and chromogenic chromID® ESBL (bioMérieux, Marcy l’Etoile, France) media and incubated at 35 ± 1 °C at least 48 h. All isolates were identified with standard microbiological procedures using the VITEK 2 system (Vitek GN-ID, bioMérieux) or MALDI-TOF (Bruker Daltonics, Bremen, Germany). Susceptibility testing was performed with the VITEK 2 system (Vitek AST-N248). EUCAST breakpoints were used for interpretation (v. 7.1 in 2017 and v. 8.0 in 2018). At least one isolate per patient and per phenotype (antibiotic susceptibility testing) was stored in a 30%-glycerol stock at − 20 °C. During the study period, *E. cloacae* complex isolates from screening and clinical specimens were taken into account.

### Band-based genotyping

Strain relatedness was assessed by a combination of random amplification of polymorphic DNA (RAPD) using the primer ST272 and repetitive-element PCR (rep-PCR) using two primers: ERIC-1 and ERIC-2 [14, 15]. In the text this method is referred to as RAPD. Isolates differing by one or more bands in at least one primer assay were assigned to distinct types (RAPD type). Every single new RAPD genotype was included in every new run.

Genotyping was additionally carried out by pulsed-field gel electrophoresis (PFGE) on all isolates after *Xba*I (New England BioLabs, Ipswich, MA, USA) restriction under the following conditions: 6 V/cm for 20 h with pulse times of 5–50 s at 14 °C. To overcome degradation bacteria were heated in EDTA and thiourea was added to the buffer [16]. The strain relatedness was calculated with GelCompar II version 6.0 software (Applied Maths NV, Sint-Martens-Latem, Belgium) and in accordance with the Tenover et al. criteria [17].

### Fourier-transform infrared (FTIR) spectroscopy

Retrospectively, all isolates were analysed by FTIR spectroscopy. FTIR spectrum acquisition and analysis was performed using an IR Biotyper System (Bruker Daltonik, Bremen, Germany) running the IR Biotyper software (version 1.5) as previously described [18]. A cut-off value of 0.77 was chosen for cluster attribution. Additionally, to improve the discrimination and clustering of FTIR spectra in our dataset we used an artificial neural network (ANN) established by Vogt et al. [18] The concordance of FTIR clustering in comparison to WGS genotyping was determined by calculation of the adjusted rand index (ARI) [19] using the online tool at www.comparingpartitions.info.

### Genome sequencing, assembly and analysis

Genomic DNA was extracted for WGS analysis, using the UltraClean Microbial DNA isolation kit (MOBIO Laboratories Inc., Carlsbad, CA, United States) or DNeasy Ultraclean Microbial Kit (Qiagen, Venlo, Netherlands). DNA library preparation was performed with the TruSeqNano DNA LT or HT Kit (Illumina, San Diego, CA, United States) and sequenced on an Illumina MiSeq (Illumina, San Diego, CA, United States) or Illumina Nextseq (Illumina, San Diego, CA, United States) sequencer. Assembly of sequence reads was performed as previously described by Vogt et al. using the A5 pipeline (version 20,140,604) and SPAdes (version 3.7.0) [18, 20, 21]. Core genomes for phylogenetic analysis were calculated using Spine (version 0.1.2) [22]. Prophage regions were investigated using PHASTER2 and removed using a customized script of the A5 pipeline [23]. SNP calling was performed by mapping high-quality sequencing reads previously generated by Trimmomatic (version 0.35) to the core genome using BioNumerics 7.6 (Applied Maths/bioMérieux, Sint-Martens-Latem, Belgium) with default settings [24].

The traditional multilocus sequence type was assessed by the CGE MLST platform (software version 2.0.4, database version 2.0.0) [25]. Acquired resistance genes on assembled sequences were identified by ResFinder (version 4.1; threshold of 98% identity and minimum length of 60%) [26]. Afterwards WGS-based species identification with the assembled genomes was performed using JSpeciesWS (version 3.6.1) [27]. Sequence reads of the strains have been deposited as a project at the European Nucleotide Archive under the project accession number PRJEB46479 (samples ERS6676481 through ERS6676512).

### Infection prevention and control (IPC) analysis

Bacterial colonizations and infections were considered as community-acquired (including acquisition at birth), if the collection of the specimen or the start of infection occurred on or before the 2nd day after admission/birth. Thereafter, bacterial colonizations and infections were defined as hospital-acquired. Transmission analysis was based on epidemiological data (direct room or ward contact, and/or documented care by the same staff) and genetic data. Transmission events were defined as proven if isolation of genetically-related isolates from two patients who were on the same ward at the same time (patient-to-patient transmission) or in the same room with a maximum time interval of one week (room-to-patient transmission). Hospital-acquired infections were classified according to the CDC/NHSN definitions [28]. Patients without related signs of infection were considered to be colonized. Standard and contact precautions (barrier nursing, use of gowns and gloves) were applied for every patient colonized with a third-generation cephalosporin-resistant *E. cloacae* complex (3GCR-EC). In case of additional ciprofloxacin or carbapenem resistance, patients were preferably isolated in a single room. Alternatively, strict contact precautions were applied. Additional interventions were performed throughout the outbreak period (training of the healthcare workers, hand hygiene compliance observations, extensive environmental sampling (surfaces, medical devices etc.), and intensified twice daily cleaning and disinfection). During the outbreak period clinicians were advised to use carbapenems in case of neonatal sepsis as this is the optimal choice to treat *E. cloacae* complex.

## Results

### Isolate and patient characteristics

Between September 2017 and March 2018, 32 patients were found to be colonized with *E. cloacae* complex. Out of these patients eight infants were twins (four sets). All except one patient were preterm neonates (gestational age < 37 weeks) with a median (range) gestation age of 26 + 0 (23 + 1–38 + 2) weeks and a median (range) birth weight of 905 (355–3000) g. 22 patients were of very low birth weight (< 1500 g). The median (range) age and length of stay at first isolation were 18 (4–109) days and 18 (1–67) days, respectively. All isolates but one were hospital-acquired. One patient developed a primary bloodstream infection with *E. cloacae* complex that was successfully treated (empirically with cefotaxime and definite therapy with meropenem); all other patients were considered as colonized.

Overall, 18 patients carried a third-generation cephalosporin-susceptible *E. cloacae* complex (3GCS-EC) isolate, five patients a 3GCR-EC and nine patients *E. cloacae* complex isolates displaying both resistance types. 24 first isolates and 8 follow-up isolates were available for further genotyping analysis. One follow-up isolate was collected after the study period from patient ID27. Relevant microbiological, epidemiological, resistome, and genotyping data are displayed in Table 1.

**Table 1:** Characteristics of the 32 *E. cloacae* complex isolates from the study period, ordered by WGS clusters and patient numbers

Patient no. (set of twins no.)	Isolate no.	Date (mo-yr)	Ward	Sample type	Species	Antibiotic resistance	Reistance genes	WGS cluster type	RAPD type	PFGE type	MLST	FTIR type
ID01	10,457	Sep-17	NICU	perianal	*E. hormaechei* subsp. *steigerwaltii*	CTX-R, ERT-I	*bla*_ACT-15_, *fosA*	1	1	A	50	A
ID01	10,502	Sep-17	NICU	oropharyngeal	*E. hormaechei* subsp. *steigerwaltii*		*bla*_ACT-15_, *fosA*	1	1	A	50	A
ID02 (1)	10,501	Sep-17	NICU	nasal/oropharyngeal	*E. hormaechei* subsp. *steigerwaltii*		*bla*_ACT-15_, *fosA*	1	1	A	50	A
ID03	10,500	Sep-17	NICU	nasal/oropharyngeal	*E. hormaechei* subsp. *steigerwaltii*	GEN-R	*bla*_ACT-15_, *fosA*	1	1	A	50	A
ID03	10,504	Sep-17	NICU	nasal/oropharyngeal	*E. hormaechei* subsp. *steigerwaltii*	CTX-R	*bla*_ACT-15_, *fosA*	1	1	n.d	50	A
ID05	10,505	Sep-17	NICU	perianal	*E. hormaechei* subsp. *steigerwaltii*	CTX-R, ERT-I, GEN-R	*bla*_ACT-15_, *fosA*	1	1	n.d	50	B
ID05	10,481	Sep-17	NICU	nasal/oropharyngeal	*E. hormaechei* subsp. *steigerwaltii*	CTX-R, ERT-R	*bla*_ACT-15_, *fosA*	1	1	A	50	A
ID06 (1)	10,503	Sep-17	NICU	oropharyngeal	*E. hormaechei* subsp. *steigerwaltii*	GEN-R	*bla*_ACT-15_, *fosA*	1	1	A	50	A
ID06 (1)	10,668	Oct-17	PICU	nasal/oropharyngeal	*E. hormaechei* subsp. *steigerwaltii*	CTX-R	*bla*_ACT-15_, *fosA*	1	1	n.d	50	A
ID07	10,593	Oct-17	PICU	nasal/oropharyngeal	*E. hormaechei* subsp. *steigerwaltii*	GEN-R	*bla*_ACT-15_, *fosA*	1	1	A	50	A
ID07	10,721	Oct-17	PICU	nasal/oropharyngeal	*E. hormaechei* subsp. *steigerwaltii*		*bla*_ACT-15_, *fosA*	1	1	n.d	50	A
ID15	10,832	Nov-17	PICU	nasal/oropharyngeal	*E. hormaechei* subsp. *steigerwaltii*	CTX-R, ERT-R	*bla*_ACT-15_, *fosA*	1	1	A	50	A
ID16	10,833	Nov-17	PICU	nasal/oropharyngeal	*E. hormaechei* subsp. *steigerwaltii*		*bla*_ACT-15_, *fosA*	1	1	A	50	A
ID04	10,506	Sep-17	PICU	central line	*E. hormaechei* subsp. *hoffmannii*		*bla*_ACT-14_, *fosA*	2	2	B	168	A
ID08	10,539	Oct-17	NICU	tracheal secretions	*E. hormaechei* subsp. *hoffmannii*		*bla*_ACT-14_, *fosA*	2	2	B	168	A
ID10	10,720	Oct-17	NICU	oropharyngeal	*E. hormaechei* subsp. *hoffmannii*		*bla*_ACT-14_, *fosA*	2	2	B	168	A
ID12	10,722	Nov-17	NICU	perianal	*E. hormaechei* subsp. *hoffmannii*		*bla*_ACT-14_, *fosA*	2	2	B	168	A
ID14	10,834	Nov-17	NICU	oropharyngeal	*E. hormaechei* subsp. *hoffmannii*		*bla*_ACT-14_, *fosA*	2	2	B	168	A
ID17	10,886	Dec-17	NICU	perianal	*E. hormaechei* subsp. *hoffmannii*		*bla*_ACT-14_, *fosA*	2	2	B	168	A
ID18 (2)	10,909	Dec-17	PICU	perianal	*E. hormaechei* subsp. *hoffmannii*	CTX-R	*bla*_ACT-14_, *fosA*	2	2	B	168	A
ID19 (2)	10,940	Dec-17	NW	perianal	*E. hormaechei* subsp. *hoffmannii*	CTX-R, ERT-R	*bla*_ACT-14_, *fosA*	2	2	B	168	A
ID21 (3)	10,995	Dec-17	NICU	perianal	*E. hormaechei* subsp. *hoffmannii*		*bla*_ACT-14_, *fosA*	2	2	n.d	168	A
ID22 (3)	10,994	Jan-18	NICU	oropharyngeal	*E. hormaechei* subsp. *hoffmannii*	CTX-R	*bla*_ACT-14_, *fosA*	2	2	B	168	A
ID13	10,749	Nov-17	PICU	tracheal secretions	*E. hormaechei* subsp. *oharae*		*bla*_ACT-7_	3	3	C	1086	H
ID13	11,155	Feb-18	PICU	tracheal secretions	*E. hormaechei* subsp. *oharae*	CTX-R, ERT-R, IPM-I, MEM-R	*bla*_ACT-7_	3	3	C	1086	E
ID20	10,938	Dec-17	PICU	perianal	*E. hormaechei* subsp. *steigerwaltii*	CTX-R	*bla*_ACT-7_, *fosA*	4	4	D	116	C
ID24	11,062	Jan-18	PICU	eye	*E. hormaechei* subsp. *hoffmannii*		*bla*_ACT-14_, *fosA*	5	5	E	104	D
ID24	11,115	Jan-18	PICU	perianal	*E. hormaechei* subsp. *hoffmannii*	CTX-R	*bla*_ACT-14_, *fosA*	5	5	E	104	D
ID26	11,183	Feb-18	NICU	perianal	*E. cloacae* subsp. *cloacae*		*bla*_CMH-3_, *fosA*	6	6	F	477	F
ID27 (4)	11,227	Feb-18	PICU	nasal/oropharyngeal	*E. hormaechei* subsp. *hoffmannii*		*bla*_ACT-14_, *fosA*	7	7	G	278	G
ID27 (4)	12,015	Aug-18	PICU	groin	*E. hormaechei* subsp. *hoffmannii*	CTX-R, CIP-I, MXF-R	*bla*_ACT-14_, *fosA*	7	7	G	278	B
ID29 (4)	11,229	Feb-18	PICU	eye	*E. hormaechei* subsp. *hoffmannii*		*bla*_ACT-14_, *fosA*	7	7	G	278	G

Antibiotic resistance (I, intermediate; R, resistant): CTX, cefotaxime; ERT, ertapenem; IPM, imipenem; MEM, meropenem; GEN, gentamicin; CIP, ciprofloxacin; MXF, moxifloxacin; Wards: NICU, neonatal intensive care unit; PICU, paediatric (and neonatal) intensive care unit; NW, neonatal ward; n.d., not determined

### Genotyping and transmission analysis

Based on conventional genotyping by RAPD and PFGE, we were able to show two main clonal clusters of *E. cloacae* complex isolates: PFGE type A/RAPD type 1 cluster containing eight patients (including one set of twins) and PFGE type B/RAPD type 2 cluster containing 10 patients (including two sets of twins). The other clusters contained isolates from the same patient (PFGE type C/RAPD type 3 and PFGE type E/RAPD type 5) or from twins (PFGE type G/RAPD type 7). Isolates 10,938 and 11,183 were the sole members of PFGE type D/RAPD type 4 and PFGE type F/RAPD type 6, respectively. All RAPD/PFGE clusters were confirmed by whole genome sequencing using a single-nucleotide polymorphism (SNP)-based phylogeny (Table 1). Intra-cluster variability was at most one SNP, whereas inter-cluster SNP difference was at least 180 SNPs (Fig. 2). Isolate 11,183 exhibited the largest genetic distance to all other isolates. Overall, all twins were colonized with the same clone and all patients from whom two resistance types were recovered (3GCR-EC and 3GCS-EC) carried one clone.

**Fig. 1:**
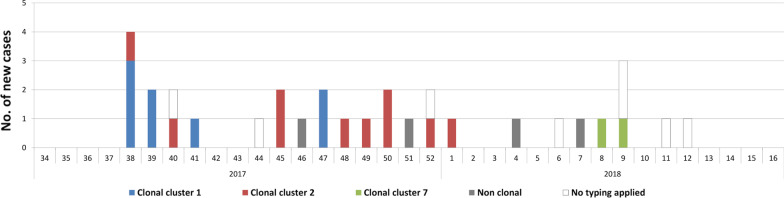
Epidemiological curve of new cases with *E. cloacae* complex per calendar week (only first isolate of *E. cloacae* complex from each patient). Only clusters containing more than one patient are shown in different colours, all other isolates are “non clonal” (no clonal relationship to other patients)

The epidemiological and WGS typing data of the *E. cloacae* complex surveillance are displayed in Fig. 1.

**Fig. 2:**
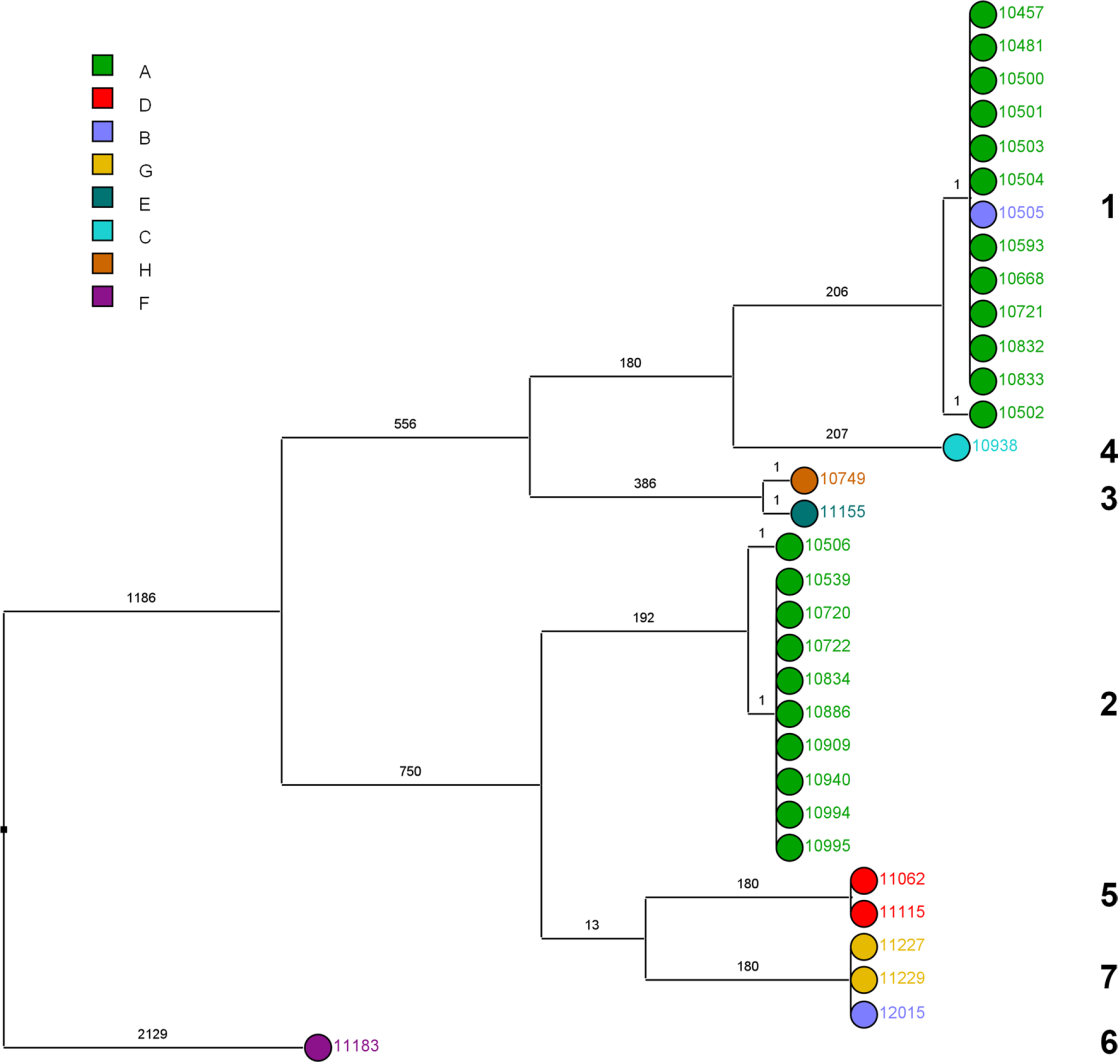
Genomic and spectral clustering of 32 *E. cloacae* complex isolates from 24 patients. SNP-based clustering of *E. cloacae* complex isolates. Values on the branches indicate the number of SNPs with logarithmic scaling of the branch length. Numbers on the right show the assigned SNP cluster types. The colour coding indicates the assigned FTIR spectroscopy cluster of the isolates

FTIR spectroscopy-based clustering displayed relatively poor correlation with the SNP-based clustering WGS reference (Fig. 2). Infrared spectroscopy was not able to differentiate WGS clonal clusters 1 and 2. First and follow-up isolates from Patient ID13 and ID27 were not classified as being clonally related. The ARI value for comparison of FTIR spectrum-based clustering (using a similarity cut-off value of 0.77) and SNP-based clustering was 0.436 (when a value of 1 represents complete concordance of the resulting clusters by each method). The spectrum-based clustering did not change, when an Artificial Neural Network was employed for assessing the spectral similarity.

Analysing spatiotemporal links, we were not able to identify index patients admitted with the clonal clusters. By conventional epidemiology, all other transmission events within clonal cluster 1, 2 and 7 were confirmed as “proven”, except for patient ID04 (isolate 10,506) from cluster 2 who had no epidemiological link to the other patients. Although most proven transmissions occurred within the NICU and all patients stayed on the NICU, three patients in clonal cluster 1 must have acquired the respective clone afterwards on the PICU from a patient transferred from the NICU. Analysing the epidemiology of the eight non-genotyped isolates from the study period only one patient could be linked to the clonal cluster 1 and another one to the clonal cluster 2.

### Further whole genome sequencing analysis

In-silico species identification revealed four different (sub-)species of *E. cloacae* complex: *E. hormaechei* subsp. *steigerwaltii*, *E. hormaechei* subsp. *oharae*, *E. hormaechei* subsp. *hoffmannii* and *E. cloacae* subsp. *cloacae* respectively (Table 1). Genetic in silico search for *bla* genes causing third-generation cephalosporin-resistance displayed AmpC beta-lactamase genes in all isolates encoding different ACT-types in 31 isolates and CMH-3 in one isolate. All isolates except two carried the *fosA* gene (Table 1).

## Discussion

In this study, we report on a molecular surveillance of *E. cloacae* complex in a perinatal centre of a tertiary care center. During the six-month study period, an increase in colonization with *E. cloacae* complex was observed in a group of preterm infants of which more than half had a very low birth weight; at first two dominant clones emerged followed by an increase of polyclonal isolates. Throughout the hospital stay preterm neonates are a vulnerable group prone to colonization with common Gram-negative organisms such as *E. cloacae* complex. Actually, *Enterobacter* species are one of the dominant bacterial organisms of the intestinal microbiome development in preterm low birth weight infants compared to term infants [5]. In a study, colonization with *E. cloacae*, the most common mucosal colonizer, was associated with the gestational age [6]. However, colonization with *E. cloacae* complex can serve as a source for infections or transmissions. Various outbreaks on neonatal intensive care units are described in the literature [29–33]. Thus, active routine microbiological surveillance screening can guide the antimicrobial empirical treatment and infection control strategies.

Whether hospital-acquired *E. cloacae* complex colonizations in two or more patients are independent acquisitions of different strains or transmission events of one clone is difficult to assess based on the microbiological report alone. It is known that *E. hormaechei* and *E. cloacae* are the most frequently isolated species from this group in human clinical specimen [1]. However, species identification of the different *E. cloacae* complex species cannot be routinely performed by most laboratories and demands more laborious molecular analysis. First reports that identification of different *E. cloacae* complex species with MALDI-TOF is feasible are promising [34]. Hence, if direct and reliable identification to the species or subspecies level is possible, this will lead to a better epidemiological understanding. Moreover, if different species are directly detected, workload and arising expenses of bacterial typing by the IPC team can be reduced. By demonstrating the molecular detection of four different species our study supports the need for research and development especially of MALDI-TOF mass spectrometry, which is used in most German laboratories nowadays. Moreover, differentiation based on phenotypes such as the antibiotic susceptibility pattern to rule out transmission events can be misleading, as clonal outbreaks with one clone displaying different phenotypes are quite common in *E. claoacae* complex [32]. The most important intrinsic mechanism of third-generation cephalosporin resistance in *E. cloacae complex* is de-repression of AmpC β–lactamases (e.g., of the ACT-type, found in nearly all isolates of this study) [1, 4]. When a patient was colonized with bacterial isolates exhibiting different resistance patterns (third-generation cephalosporin resistance or susceptibility) over time in this study, genotyping showed that the isolates were genetically highly-related, suggesting mutational evolution probably by antibiotic selection pressure.

Overall, typing methods are crucial to confirm or rule out transmission events. Low-discriminatory methods can lead to an overestimation of an outbreak and to unnecessary IPC interventions [33]. The preferred method should be selected based on its discriminatory power, its turnaround time and techniques available on site. Other constraints are reproducibility, costs or hands-on time [35, 36]. In this study, we applied typing methods of moderate to high discriminatory power (RAPD/rep-PCR, FTIR spectroscopy, MLST and PFGE) and compared those with WGS, which serves as the current gold standard of bacterial typing. All methods are well described for *E. cloacae* complex except for FTIR spectroscopy. This technique was only recently studied on a selection of *E. cloacae* complex isolates with overall satisfying results [18, 37]. Vogt et al. showed good concordance with SNP-based clustering (ARI = 0.818) [18]. Distinct bacterial cell structures are targeted by the typing methods applied in this study: unspecified genomic sequences (RAPD), repetitive genetic elements (rep-PCR), genomic restriction sites (PFGE), (mostly) polysaccharides (FITR), several housekeeping genes (MLST) or the whole genome (WGS). Comparative analysis of the isolate collection of this study showed good concordance between the conventional methods (RAPD/rep-PCR, PFGE and MLST) and WGS whereas the discriminatory power of FTIR was only moderate. Other studies showed more discriminatory results using FTIR for outbreak investigation [18, 37]. FTIR depends much more on the bacterial phenotype, the overall cellular composition and growth conditions than the other methods [18]. We also experienced that mucoid bacteria might be difficult to evaluate by this technique (e.g. patient ID13 carrying one mucoid and one non-mucoid type).

RAPD and rep-PCR both banding patterns typing methods can differ in discriminatory power. For example, Steffen et al. showed little discriminatory power compared to all other method used, but applied only one arbitrary primer variant [33]. However, these methods have the advantage that they are inexpensive and quick and can be a reliable tool if the information of several primers is combined and if reference strains are run as shown with different species and settings [38, 39]. Primers, cyclers and gel chambers needed for this method are available in most laboratories. Difficulties can arise from varying band intensities and may thus result in interpretation errors. These methods have their limits in case of complex outbreaks with many different strains involved as gels are less comparable compared to PFGE [33]. PFGE on the other hand is very labor intensive, but leads to good results in many cases [35].

WGS as the new gold standard is cost intensive and needs advanced skills of interpretation. Usage of WGS was previously described in *E. cloacae* outbreaks [40, 41]. A worldwide application of WGS is neither affordable nor necessary in most circumstances, especially to rule out transmission. At the moment, its application in real-time surveillance is limited due to costs and turnaround time. However, other information than the genotype can be obtained from the WGS data such as (sub-)species, plasmid structures or resistome.

In the clinical setting of nosocomial acquisition, reservoirs such as other humans, mostly patients, or the environment have to be identified. In general, the most common cause of nosocomial acquisition is person-to-person transmission via contaminated hands by health-care workers. Nevertheless, colonized healthcare workers are rarely described as a source of an outbreak [42]. Another source is the environment. However, we were not able to find an environmental source despite extensive efforts. No more *E. cloacae* complex isolates were detected on the NICU and PICU in the colonization screening during the three months following the study period (April–June 2018).

There are a few limitations to this study. First, we only analyzed one isolate per resistance pattern per patient. Patients colonized/infected with more than one strain of the same antibiotic resistance pattern might have remained undetected. Secondly, our conclusions cannot be generalized due to the limited number and the specific epidemic setting. Thirdly, rectal or vaginal screening of mothers recommended in the likelihood of a preterm delivery was not performed on a regular basis. Thus mother-to-child transmission could not be assessed.

## Conclusions

A molecular and infection surveillance of hospital-acquired *E. cloacae* complex based on periodic screening, conventional epidemiology, genotyping and identification to the species level revealed simultaneously occurring independent transmission events and clusters and four different species. This underlines the importance of such an extensive surveillance methodology in IPC programs especially in vulnerable patient populations such as preterm neonates.

## Data Availability

Sequence reads have been deposited at the nucleotide accession number GenBank PRJEB46479. All other data generated or analysed during this study are included in this published article.

## Abbreviations

ANN: Artificial neural network
ARI: Adjusted rand index
CDC: Centers for Disease Control and Prevention
cgMLST: Core genome multilocus sequence type
EUCAST: European Committee on Antimicrobial Susceptibility Testing
FTIR: Fourier-transform infrared
ICU: Intensive care unit
IPC: Infection prevention and control
ITS-KISS: Intensivstation-Krankenhaus-Infektions-Surveillance-System = German national nosocomial infections surveillance on intensive care units
MALDI-TOF: Matrix-assisted laser desorption/ionization-time-of-flight mass spectrometer
MDR: Multidrug-resistant
MLST: Multilocus sequence type
NEO-KISS: Surveillance System nosokomialer Infektionen für Frühgeborene auf Intensivstationen = German national neonatal infection surveillance system (for very low birth weight infants)
NHSN: National Healthcare Safety Network
NICU: Neonatal intensive care unit
PFGE: Pulsed-field gel electrophoresis
PICU: Paediatric (and neonatal) intensive care unit
RAPD: Random amplification of polymorphic DNA
rep-PCR: Repetitive-element PCR
SNP: Single-nucleotide polymorphism
WGS: Whole genome sequencing
3GCS-EC: Third-generation cephalosporin-susceptible *E. cloacae* complex
3GCR-EC: Third-generation cephalosporin-resistant *E. cloacae* complex
